# Canine parvovirus-like particles, a novel nanomaterial for tumor targeting

**DOI:** 10.1186/1477-3155-4-2

**Published:** 2006-02-13

**Authors:** Pratik Singh, Giuseppe Destito, Anette Schneemann, Marianne Manchester

**Affiliations:** 1 Center for Integrative Molecular Biosciences, The Scripps Research Institute, La Jolla, CA 92037, USA; 2 Department of Cell Biology, The Scripps Research Institute, La Jolla, CA 92037, USA; 3 Department of Molecular Biology, The Scripps Research Institute, La Jolla, CA 92037, USA; 4 Dipartimento di Medicina Sperimentale e Clinica, Università degli Studi Magna Graecia di Catanzaro Campus Universitario di Germaneto, Catanzaro, ITALY

## Abstract

Specific targeting of tumor cells is an important goal for the design of nanotherapeutics for the treatment of cancer. Recently, viruses have been explored as nano-containers for specific targeting applications, however these systems typically require modification of the virus surface using chemical or genetic means to achieve tumor-specific delivery. Interestingly, there exists a subset of viruses with natural affinity for receptors on tumor cells that could be exploited for nanotechnology applications. For example, the canine parvovirus (CPV) utilizes transferrin receptors (TfRs) for binding and cell entry into canine as well as human cells. TfRs are over-expressed by a variety of tumor cells and are widely being investigated for tumor-targeted drug delivery. We explored whether the natural tropism of CPV to TfRs could be harnessed for targeting tumor cells. Towards this goal, CPV virus-like particles (VLPs) produced by expression of the CPV-VP2 capsid protein in a baculovirus expression system were examined for attachment of small molecules and delivery to tumor cells. Structural modeling suggested that six lysines per VP2 subunit are presumably addressable for bioconjugation on the CPV capsid exterior. Between 45 and 100 of the possible 360 lysines/particle could be routinely derivatized with dye molecules depending on the conjugation conditions. Dye conjugation also demonstrated that the CPV-VLPs could withstand conditions for chemical modification on lysines. Attachment of fluorescent dyes neither impaired binding to the TfRs nor affected internalization of the 26 nm-sized VLPs into several human tumor cell lines. CPV-VLPs therefore exhibit highly favorable characteristics for development as a novel nanomaterial for tumor targeting.

## Background

Conventional chemotherapy for treating cancer is non-selective and therefore associated with toxic side effects, limiting a drug's therapeutic index [1-4]. Targeted delivery of drugs is ideal in order to enhance therapeutic benefit as well as reduce systemic toxicity. Recently the development of novel methods to achieve specific tumor targeting has received significant focus [5,6]. Strategies investigated towards this goal include "smart" tissue-specific particles such as liposomes [7], antibodies [8,9], viral particles [10-12] and dendrimers [13] that are comprised of targeting moieties and cytotoxic drugs.

Currently virus-based nanoparticles (VBNPs) are being extensively investigated for nanobiotechnology applications [12,14]. Many viral particles are in the nanometer size range and are naturally uniform in size because of the structural constraints on capsid assembly. An increasing number of three-dimensional virus structures known to atomic resolution paved the way for derivatization of VBNPs with dyes, metals, peptides, proteins, and small molecules and is being explored for generating novel nanomaterials. In the last decade several VBNPs have been examined for diverse applications such as templates for material synthesis, platforms for polyvalent display, electronic components, and drug targeting [14-19]. Typical characteristics for a VBNP platform qualification include knowledge about its crystal structure, ability to produce in substantial quantities, stability in a wide range of pH, and suitability for genetic manipulation as well as chemical bioconjugation. Viruses and virus-like particles (VLPs) that have been developed for nanotechnology purposes include bacteriophages (M13 and MS2 [16,20]), plant viruses (cowpea mosaic virus (CPMV), cowpea chlorotic mottle virus (CCMV) and tobacco mosaic virus(TMV) [15,18,21,22]), an insect virus (flock house virus [23]), and animal viruses (adenovirus, polyoma virus [24,25]). While infectious plant viral particles can be produced in large quantities, generating substantial amounts of most animal viruses in cell culture systems is not economical. However, production of VLPs in adequate quantities has been achieved by expression of virus capsid proteins in heterologous systems (insect cells, yeast, and bacteria). VLPs are generally found to be structurally identical to native virus particles and more importantly are non-infectious. Viral particles are also being explored as tumor targeting agents. Since most of the established VBNPs do not have any specificity for tumor cells and therefore need to be either genetically or chemically modified in order to achieve targeted delivery. These targeting strategies are typically not as efficient when compared to the natural cell receptor targeting potential of a virus.

**Figure 1:**
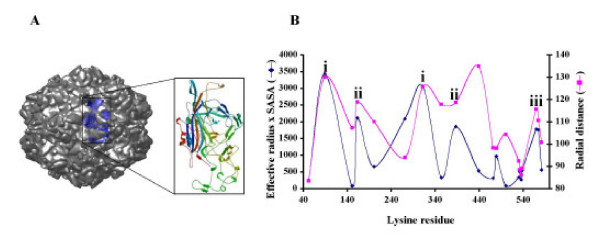
**CPV Capsid and subunit organization**. The 2CAS model of CPV was downloaded from the VIPER database. The expanded inset shows a single VP2 subunit ribbon diagram with N-terminus in blue and C-terminus in red. **B. Accessible surface lysines profile of CPV capsid**. Data shown was downloaded from VIPER database. Lysine residues in the VP2 subunit are shown on X-axis (total of 20 per subunit) and the effective radius multiplied by the solvent accessible surface area (SASA) is shown in blue on Y-axis on the left side. The radial distance of each residue is also shown on Y-axis in magenta. Coinciding high values on Y-axis suggest residues that are (i) highly accessible, (ii) moderately accessible and (iii) accessible to a lesser extent.

In this study we characterized canine parvovirus (CPV)-VLPs as a potential nanomaterial for tumor targeting purposes. CPV, a viral pathogen of canids (dogs) belongs to the family *parvoviridae *[26]. The infectious agent is an icosahedral (T = 1), non-enveloped virus encapsidating a single stranded DNA of about 5 kb and shows an average diameter of 26.4 nm. The viral DNA encodes three polypeptides VP1, VP2 and VP3 that are generated by alternative splicing of viral mRNA. The crystal structure of the virion revealed that a full (DNA-containing) capsid is composed of 60 subunits, primarily of the VP2 subunits (64 kDa) and a few VP1 and VP3 subunits. While empty capsids contain mostly VP2 subunits along with a minor amount of VP1 subunits but lack VP3 subunits [26]. Each subunit is made up of a central 'jelly roll' anti-parallel β-barrel core with elaborate loops between the β-strands (Figure 1A) [26]. Generation of CPV-VLPs in both mammalian cells and insects cells by expressing only the VP2 gene has been described previously [27]. The transferrin receptor (TfR) on canine cells serves as a cellular receptor for the native CPV [28]. Interestingly, infectious CPV particles were also found to bind and enter human cells utilizing TfRs, however, subsequent steps in the replication cycle did not appear to be supported [28].

Transferrin is a circulatory iron carrier protein that is in great demand particularly during cellular growth and proliferation [29]. Since iron is also required by rapidly dividing cancerous cells, a significant upregulation of transferrin receptor expression is seen in a wide variety of tumor cells. Indeed, analysis of TfR expression revealed approximately 10^5 ^or more receptors per cell in several breast cancer cell lines (including MDA-MB-231) [30], HeLa (human cervical carcinoma) [31], HT-29 (human colon carcinoma cells) [32], K562 (human erythroleukemia cells) [31,33] and pancreatic tumor cells [34] compared to a few or often undetectable TfR levels in normal cells [35]. Therefore tagging a drug or image contrast agent to transferrin for specific delivery to tumor cells emerged as a promising strategy and is being widely explored for tumor-targeted delivery [35,36]. Thus CPV-VLPs that bind to human TfRs may hold an advantage over other viral nanoparticles for tumor-specific delivery.

In this study we examined the suitability of CPV-VLPs for tumor-targeting applications such as chemical modification with small molecules and capability to deliver those molecules to the tumor cells. CPV-VLPs produced in a baculovirus expression system were analyzed for the accessibility and chemical reactivity of capsid surface-exposed lysines for derivatization with fluorescent dye molecules. Binding and internalization of dye-derivatized CPV-VLPs in various human tumor cells was investigated.

## Results and discussion

For generation of CPV-VLPs, a recombinant baculovirus expressing the full length CPV-VP2 gene (encoding 584 amino acids) under the control of the polyhedrin promoter was utilized to infect insect cells as described previously [27]. In this study, however, instead of *Sf*-9 cells we utilized insect *T.ni *cells for production of VLPs as they are known for enhanced protein production. *T.ni *cells infected with recombinant baculovirus were harvested at different time points (daily from between one through five days) to optimize the yield of CPV-VLPs. An incubation length of 72–96 hrs post-infection was found to be optimal for maximizing the yield. The VLP yields ranged from 0.5 to 2 mg/ liter of infected *T.ni *cell culture. Harvest of cells before 48 hrs or after 5 days post-infection reduced the VLP yield to less than 50% of a 3–4 day harvest. While early harvest suffered from inefficient infection, late harvest presumably leads to cell lysis releasing VLPs that seem to be vulnerable to cell- or baculovirus-derived proteases (data not shown). It was previously shown that although a large amount of CPV-VP2 protein could be expressed within S*f*-9 insect cells, a portion of VP2 fails to assemble into VLPs [27]. During a native parvovirus infection, approximately 50% of the assembled capsids were found to be empty (non-infectious) and composed primarily of VP2 subunits with a few VP1 subunits [26]. Since the VP2 protein alone was expressed in the current study, a co-expression of VP2 and VP1 in the baculovirus expression system may enhance the assembly process and thereby improve the yield of CPV-VLPs. Closely related porcine parvovirus-VLPs appear to assemble more efficiently than CPV in the baculovirus expression system as their yields were substantial, approximately 120 mg/liter of culture in a bioreactor [37]. VLPs of polyoma virus [38], hepatitis B virus surface antigen [39], hepatitis delta virus [40] and CCMV [41] have also been produced in large quantities in a yeast expression system that may be useful for generating CPV-VLPs.

**Figure 2:**
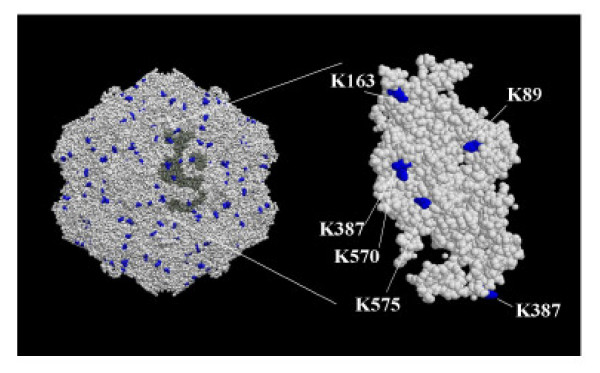
**Space filling model of surface accessible lysines on CPV capsid**. The CPV capsid model was generated with VMD software. The figure shows identified accessible lysines on CPV based upon the whole capsid (left side) and on an individual VP2 subunit (right side).

To evaluate whether CPV-VLPs could be efficiently derivatized by chemical methods as has been performed for several viral nanoparticles [14], the location of surface lysines on CPV-VLPs was identified based upon a structural model of CPV using the radial distance and solvent accessibility surface area parameters in the VIPER database as described in the methods. Based on the analyses, lysines at positions 89 and 312 are highly accessible while those at positions 163 and 387 are moderately accessible and those at positions 570 and 575 on the particle surface are accessible to lesser extent (Figure 1B). Using this model, two (maximum of 6) lysines of the twenty lysines per VP2 subunit, or 120 (maximal of 360) lysines per CPV-VLP particle could theoretically be accessible on the capsid surface for bioconjugation. However, the reactivities are known to vary from their predicted accessibility based upon the local chemical environment of the lysine residue on the capsid surface [15]. Surface accessible lysines on the capsid and on a single subunit of VP2 are depicted in a space filling model in Figure 2. Immediately following purification, the CPV-VLPs were examined for reactivity and conjugation to the lysines by exposure to NHS-Oregon Green 488 (OG-488). In most cases, using 100 molar equivalents of OG-488 dye molecules per VP2 subunit in the VLP preparation, an average of 45 lysines/particle were addressed. Exposure to 200 molar equivalents of the dye per VP2 subunit resulted in an average of 100 derivatized lysines/particle. Further increase in the dye equivalents did not appear to enhance CPV-VLP labeling (data not shown). Initially, the virus labeling was carried out in a phosphate buffer (0.1 M potassium phosphate) similar to dye derivatization of CPMV [15]. The CPV-VLP particles, although stable in phosphate buffer, could not withstand the presence of additional 10% DMSO and a dye, which caused disassembly of the VLPs into subunits. In contrast CPMV particles are known to withstand such labeling environment [15]. Performing dye labeling of CPV-VLPs in PBSE buffer, or in phosphate buffer containing 150 mM sodium chloride stabilized the particles (data not shown).

**Figure 3:**
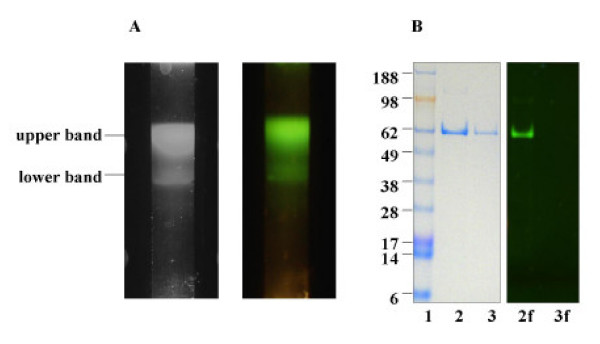
**CPV purification and characterization. A. Sucrose gradient purification**. VLPs preparation from infected cell culture lysates purified by sucrose gradient centrifugation (10–40%). Bands of CPV-VLPs that were derivatized with OG-488 are visible in the gradient just above the middle of the tube (left panel) and appear fluorescent green under a UV-light source (right panel). **B. SDS-PAGE analyses**. The purified VLPs were subjected to electrophoresis in 4–12% Bis-tris gel and stained with SimplyBlue (Invitrogen) to reveal the proteins (left panel). The Seeblue plus protein molecular weight standards in kDa (Invitrogen) are indicated on the side of the gel picture (lane 1). Lanes 2 and 3 contain protein from CPV-VLPs derivatized with OG-488 and CPV-VLPs respectively. Prior to staining, the gel (right panel) visualized with a UV-light source showed a fluorescent 62 kDa band in the lane of OG-488 derivatized CPV-VLPs (lane 2f) and lacked any fluorescent bands in the native CPV-VLPs (lane 3f).

To characterize the CPV-VLPs from the infected *T.ni *cell culture, particles were centrifuged in a 10–40% sucrose gradient. The VLPs formed two bands visible about the middle of the tube (data not shown). A hazy smeared upper band presumably represented a mixture of empty particles (lacking nucleic acids) and particles with variable amounts of nucleic acids. The lower faint band we hypothesized was comprised of particles with a definite amount of randomly packaged cellular nucleic acid material. The proportions of these bands varied greatly over each preparation. The packaging of non-specific cellular nucleic acids into VLPs during baculovirus expression has been described [42]. The smeared VLP bands in the gradient also suggested VLP preparation has packaged variable amounts of nucleic acids that are most likely random cellular RNA since the particles assemble in the cytoplasm (data not shown). Gradient purified VLP preparation was collected and analyzed by SDS-PAGE for presence of viral coat protein and to evaluate sample purity. The gel revealed a 62 kDa protein corresponding to the known molecular weight of CPV-VP2 protein (Figure 3B) with no obvious degradation products or impurities. The difference in VP2 gene product to an expected 64 kDa is presumed to be due variation in gel mobilities of proteins. Similar to unlabeled particles, CPV-VLP particles labeled with the dye OG-488 when separated on the gradient revealed a smeared top band and another band about the middle of the gradient, similar to that of a native CPV-VLP preparation (Figure 3A left panel). The band appeared fluorescent when exposed to a UV- light source (Figure 3A, right panel). Dye-derivatized CPV-VLPs, when analyzed on SDS-PAGE showed a fluorescent band upon exposure to a UV-light source that migrated at 62 kDa. No difference was observed in the mobility of the dye-derivatized CPV-VP2 subunit protein compared to native CPV-VP2 subunit protein under the SDS-PAGE conditions used, as expected since there are an estimated 1 to 2 molecules of OG-488 dye per VP2 subunit (with approximate increase of about 0.6 to 1.2 kDa; Figure 3B).

**Figure 4:**
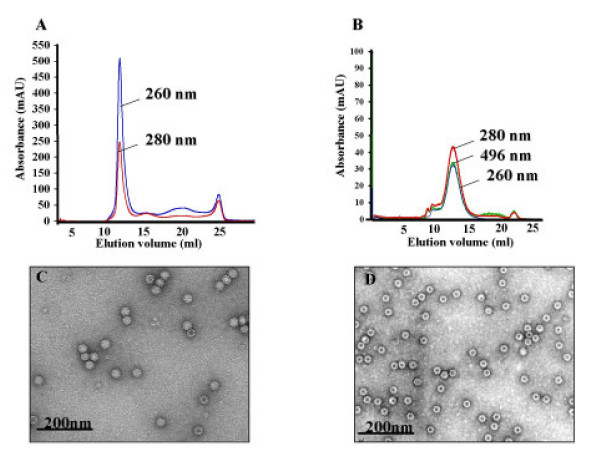
**Capsid stability and morphology of CPV-VLPs. A and B. Size exclusion chromatography (SEC) of CPV-VLPs**. Sucrose gradient purified samples were passed through a Superose6 size exclusion column. Absorbance values recorded at 260 nm (for nucleic acids), 280 nm (for protein) and 496 nm (for OG-488 dye) are shown on the y-axis. The elution profile from the column in ml is shown on x-axis. Panel A shows SEC of freshly purified CPV-VLPs and panel B shows SEC of CPV-VLPs labeled with OG-488 dye following 1 week of storage at 4°C. **C and D. Electron micrographs of CPV-VLPs**. CPV-VLPs were deposited onto carbon-coated copper grids and stained with uranyl acetate. The micrographs of (C) full capsids in a freshly purified CPV-VLPs preparation and (D) empty capsids in CPV-VLPs sample after 1 week of storage are shown. Both micrographs were taken at a nominal magnification of 60,000×.

The sucrose gradient-purified particles were analyzed by size exclusion chromatography (SEC) for elution volume and absorbance indicative of particle size, intactness and packaged nucleic acid material. SEC of a freshly purified VLP preparation revealed that the absorbance at 260 nm was high compared to absorbance at 280 nm (Figure 4A) suggestive of packaged nucleic acids. With a flow rate of 0.4 ml/min in PBSE buffer (pH 7.4), the particles had elution volume of 11–13 ml on the SEC column. Cowpea mosaic virus particles (approximately 31 nm in diameter) that are routinely used our laboratory showed an elution of 8 to 10 ml in PBSE buffer (data not shown) on the same column suggested that the CPV-VLPs (26 nm-sized) are smaller in size and intact. Disassociated or unassembled subunits and other contaminant proteins showed elution volumes greater than 15 ml. Surprisingly, containment of nucleic acid material within CPV-VLPs was transitory, as the particles were found to be empty after one to two days of storage at 4°C. After a week of storage at 4°C the particles exhibited a lower absorbance at 260 nm indicating lack of nucleic acid material (Figure 4B). Presumably the packaged nucleic acid was hydrolyzed. Finding entirely empty particles immediately following purification was also not uncommon. Although empty, the CPV-VLPs were found to be quite stable in PBSE buffer after several months of storage at 4°C without showing any signs of disassembly. The particle intactness could be confirmed over the SEC column and by transmission electron microscopy (TEM) (data not shown). Analyses of dye-labeled particles on the SEC at 496 nm revealed that the conjugate dye molecules are associated with the intact VLPs (Figure 4B). TEM analyses of purified VLPs supported the observation that the particles were not empty initially following purification. Figure 4C shows the CPV-VLP capsids with an electron-dense core indicating the presence of nucleic acid. In contrast, after 7 days of storage the particles have an electron-opaque core consistent with empty capsids (Figure 4D). Interestingly, expression of coat proteins from the RNA viruses, FHV coat protein [42] or tomato bushy stunt virus [43] in the baculovirus system results in VLPs containing variable amounts of cellular RNA. However, this packaged cellular RNA is not lost upon storage. Presumably, as a DNA-containing virus, the CPV capsid interior has natural affinity for viral single-stranded DNA and therefore lacks the capability to retain any of the non-specifically packaged RNA, resulting in empty particles eventually.

Previous studies have revealed that the native CPV utilizes canine as well as human TfRs to internalize and reach the endosomes in cells [28]. Detailed analyses of CPV capsid revealed that the Asn residues at positions 93 and 300 on the three fold spike are important in binding to the canine TfRs. Additionally, several residues in the shoulder region (Gly 299, Lys 387, Ala 300, Thr 301, and val 316) also appear to play a role in binding [44]. Based on the CPV-capsid modeling (Figure 1B) the Lys residues at positions 89 and 312 are the most solvent accessible and therefore more likely to be derivatized. In our bioconjugation experiments, attachment of dyes to the Lys 387 residue in some of the subunits cannot be ruled out. However, the role of these residues in CPV binding specifically to human TfRs has not been determined.

**Figure 5:**
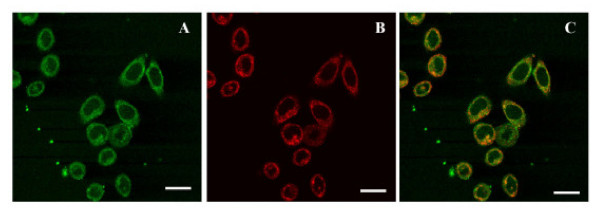
**Binding and internalization into of CPV-VLPs into HeLa cells**. HeLa cells incubated with Texas red-labeled transferrin (red) and CPV-VLPs were washed and fixed. Labeled antibodies (green) were used to detect the presence of CPV-VLPs in the cells by fluorescence confocal microscopy. **(A) **CPV-VLPs are seen as green areas in the cytoplasm, **(B) **shows localization of Texas Red-transferrin (red) and **(C) **depicts merged picture showing co-localization of CPV-VLPs and transferrin in yellow. Scale bar, 25 μm.

**Figure 6:**
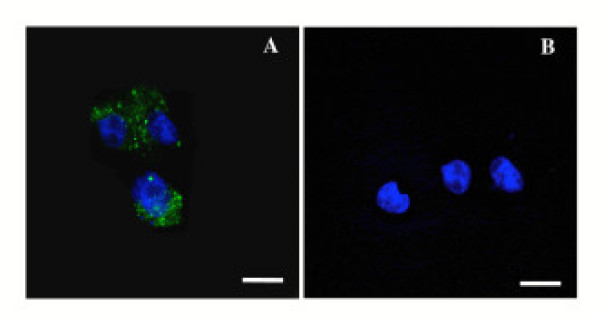
**Binding and internalization of CPV-VLPs labeled with OG-488 into transferrin receptor expressing cells**. Cells differing in level of transferrin receptor expression, TRVb1 (express TfRs) and TRVb (low or lacking TfR expression) were exposed to CPV-VLPs. Internalized dye-labeled CPV-VLPs were detected by fluorescence confocal microscopy. TOTO-3 (blue) was used for staining the nuclei. **(A) **TRVb1 cells with internalized dye-derivatized CPV-VLPs, are seen as green areas in the cytoplasm, and **(B) **TRVb cells show lack of VLP internalization. Scale bar, 25 μm.

**Figure 7:**
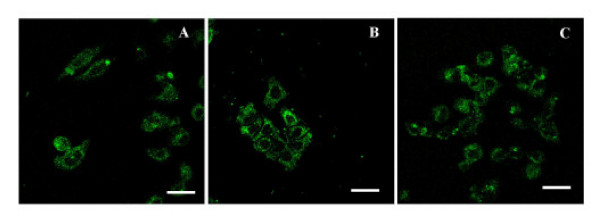
**Binding and internalization of CPV-VLPs labeled with OG-488 into tumor cell lines**. Tumor cell lines (A) HeLa, (B) HT-29 and (C) MDA-MB231 were exposed to OG488-labeled CVP-VLPs. The cells were washed, fixed and examined by confocal fluorescence microscopy for internalization of the particles. Scale bar, 25 μm.

Once it was demonstrated that CPV-VLPs could withstand chemical conjugation and remain intact following purification, the dye-labeled CPV-VLPs were investigated for their potential utility to target tumor cells. First we examined the binding and internalization of CPV-VLPs into HeLa tumor cells that over-express TfRs [31] (Figure 5). The internalization of CPV-VLP particles was fairly rapid, occurring within two hours as previously observed with native CPV [28]. Co-localization of the antibodies recognizing CPV-VLPs with Texas red-labeled transferrin confirmed that the VLPs are localized to endosomes following uptake. Confocal image analyses showed approximately 50–60 % co-localization, and the differences seen are likely due to fact that Tfn is efficiently recycled while CPV particles are diverted from endosomes to lysosomes, consistent with previous reports [28,45]. Binding to TfRs and clathrin-mediated vesicular trafficking of CPV to endosomes and lysosomes in the cells has been demonstrated previously in HeLa cells and in non-cancerous NLFK cells [28,45]. We next examined whether CPV-VLPs derivatized with OG-488 dye molecules will show a similar cell binding and internalization characteristics. To confirm the TfR specificity, the binding of OG-488-labeled CPV-VLPs to TRVb1 cells (expressing TfR), and TRVb cells (lacking or expressing very low levels of TfR) [46] was investigated. The binding and internalization of dye-labeled CPV-VLPs was observed only in the TRVb1 cells but not in TRVb cells (Figure 6) confirming that binding and internalization is TfR-mediated. Thus the TfR-specific internalization of OG-488 labeled CPV-VLPs is similar to the native CPV-virions, in agreement with an earlier report [28]. Since CPV-VLPs could efficiently enter HeLa tumor cells and the dye-labeled CPV-VLPs demonstrated TfR specificity, we then examined binding and internalization of OG-488-labeled CPV-VLPs into other human tumor cell lines that are known to over-express TfRs such as HT-29 and MDA-MB231 cells [30,31]. The CPV-VLPs derivatized with OG-488 were taken up by all three cell lines investigated within 2 hours, similar to unlabeled particles in HeLa cells (Figure 7).

Thus we have demonstrated that CPV-VLPs, derivatized with small molecules to the lysines on the capsid surface, retain their targeting for TfRs. Furthermore, CPV-VLPs can withstand the conditions required for chemical modification expanding their utility for conjugation with chemotherapeutic drugs or image contrast agents. Our future efforts are directed towards tumor targeting with dye and drug-labeled VLPs in mouse models of human cancer. Derivatization of CPV-VLPs with chemotherapeutic drugs conjugated via various kinds of endosomal cleavable linkers [8,47] is being investigated for release of the drug specifically into the tumor cell interior. While there are many kinds of nanoparticles in development for tumor targeting [6], VBNPs compared to their peers, exhibit remarkable uniformity and offer precise control over display of molecules. Achieving this level of control over spatial distribution is unparalleled with inorganic or lipid nanomaterial. However, since VLPs are proteinaceous in composition, an immune response by the host is obvious, limiting their usage for repeat administration. Utilizing multiple VLPs or employing polymer coat shielding of particles [48,49] or using altered chimeric particles [50] may address immune clearance issues.

## Conclusion

CPV-VLPs can be produced in significant quantities in the baculovirus expression system. Optimization of the expression including addition of other regions of capsid proteins or truncated versions of VP2 gene or other systems of protein expression may be useful for further improving the particle yield. Like native CPV, dye-labeled CPV-VLPs specifically bind to TfRs known to be upregulated on a variety of tumor cells. Derivatization of lysine residues on CPV-VLPs with small molecules is feasible under appropriate reaction conditions and does not interfere with the binding and internalization into tumor cells. Together these studies demonstrate the potential for development of CPV-VLPs as a novel virus-based platform for tumor targeted delivery of drugs and image contrast agents.

## Materials and methods

### Production and purification of CPV-VLPs from insect cells

Recombinant baculovirus production of CPV-VLPs has been previously described [27]. The recombinant virus was a gift of Dr. C. Parrish (Cornell University, Ithaca, New York). Initial stock preparation, plaque purification and determination of plaque forming units (pfu) was performed in *Spodoptera frugiperda *(Sf-21) cells. For large scale preparations, *Trichoplusia ni *(T.ni) cells were propagated at 27°C in EX-CELL 401serum-free medium (JRH Biosciences, Lenexa, KS) supplemented with 2 mM L-glutamine, 100 U/ml of penicillin per ml, and 100 μg/ml of streptomycin. Each liter of culture containing 1 × 10^6 ^cells/ml was infected with 15 to 20 ml of recombinant virus at a titer of 5 to 8 × 10^6 ^pfu/ml. Following incubation of infected cells at 28°C and 100 rpm in shaker flask (typically for 72 hrs), cells were harvested by centrifugation at 1500 g for 10 mins. The cells were re-suspended in 100 ml of phosphate buffered saline (PBS) containing 10 mM ethylene diamine tetra acetic acid (PBSE, pH 7.4). The cells were lysed by addition of Triton X-100 to a final concentration of 1% along with 2 mM phenylmethyl sulfonyl fluoride on ice for 10 minutes. The cell debris was pelleted by centrifugation at 10000 g for 30 mins. To the supernatant an equal volume of chloroform/butanol mixture (1:1) was added and stirred for 15 mins at 4°C. The solution was centrifuged at 10000 g for 20 mins. The aqueous supernatant was collected carefully and polyethylene glycol 8000 and sodium chloride were added to a final concentration of 8% and 400 mM respectively. The mixture after stirring for 20 mins at 4°C was centrifuged at 15000 g for 20 mins. The pellet containing the CPV-VLPs was re-suspended in 10 ml of PBSE. Following 30 minutes of mixing on a shaker at room temperature, the suspension was centrifuged at 9000 g to remove insoluble debris. The supernatant comprising of the CPV-VLPs was transferred to a tube containing 4 ml of 20% sucrose cushion in PBSE and centrifuged in a 50.2 Ti rotor (Beckman, Fullerton, CA) at 145,000 g for 3 hrs at 4°C. The pellet was resuspended in 1 ml of PBSE and then layered onto a 10–40% sucrose gradient in PBSE, and centrifuged at 207000 g for 2 hrs in a SW41 rotor (Beckman) at 4°C. Bands visible about the mid-point of tube were collected and centrifuged in 50.2Ti rotor at 145000 g for 3 hrs at 4°C. The collected pellet comprising of purified CPV-VLPs was resuspended in PBSE and stored at 4°C. Purified VLP samples were analyzed by sodium-dodecyl-sulfate polyacrylamide gel electrophoreses (SDS-PAGE), size exclusion chromatography and transmission electron microscopy. The yield of VLPs was quantitated using a Lowry protein assay kit (Pierce, Rockford, IL).

CPV-VLPs denatured in SDS-PAGE sample buffer were separated in a 4–12% bis-tris polyacrylamide gel (Invitrogen, Carlsbad, CA) by employing a 200 V constant current for 35 minutes. The protein bands were visualized by staining with Simply Blue (Invitrogen). For dye-labeled virus (see below), following electrophoresis the gel was placed on a UV-light box to visualize fluorescent bands. SEC was carried out on a Superose6 column using an AKTA explorer (Amersham-Pharmacia Biotech, Piscataway, NJ) with a flow rate of 0.4 ml/minute in PBSE buffer (pH7.4).

### CPV-VLP modeling

The CPV-VLP capsid structure (Figure 1A,B) was obtained from the virus particle explorer database (VIPER) [51]. The model shown was rendered with CHIMERA software [52]. The inset in figure 1A shows a ribbon diagram of a single VP2 protein subunit. The accessible lysines on the capsid surface were determined based on the radial distance of the residue, effective radius and solvent accessible surface area of CPV-VLPs in VIPER database that was originally determined using CHARMM software [53]. The identified surface accessible lysines were then represented in a space filling model of CPV-VLPs designed using Visual Molecular Dynamics software (VMD) [54].

### Dye labeling of CPV-VLPs

Based on previously published methods for dye labeling of the plant virus CPMV [15], CPV in PBSE was labeled with various molar equivalents of the dye, Oregon green-488 succinimidyl ester (OG-488, Invitrogen). Briefly, OG-488 dye (MW_r _= 662.5) was added to100 or 200 molar equilvalents per VP2 subunit (MW_r _= 64000) as follows. First the dye was dissolved in dimethyl sulfoxide (DMSO) and then mixed with virus in PBSE to contain not more than 10% of DMSO final concentration. A virus concentration of 2 mg/ml in PBSE was used for all dye labeling reactions. Following overnight incubation at room temperature, hydroxalamine (pH 8.5) was added to a final concentration of 1.5 M to inactivate the dye ester. The dye-labeled virus was sucrose gradient purified as described above. The collected virus band was further dialyzed with 3 exchanges against PBSE. The amount of dye conjugated onto the VLPs calculated as absorbance measured at 496 nm times the molecular weight of virus (64000 × 60) divided by the product of extinction coefficient of OG-488 dye (70000) and concentration of virus in mg/ml. VLPs derivatized with the dye were analyzed by SDS-PAGE, SEC and TEM. The binding and internalization of dye-labeled CPV-VLP in TRVb, TRVb1 and tumor cells was examined by confocal microscopy.

### Cell lines

Human tumor cell lines, HT-29, HeLa and MDA-MB231 were obtained from American Type Culture Collection (Manassas, VA). HT-29 was maintained in Leibovitz medium (Invitrogen) while HeLa and MDA-MB231 were cultured in modified DMEM (Invitrogen). Chinese hamster ovarian cells TRVb (negative for transferrin receptor expression) and TRVb1 (derived from TRVb cells containing an expression plasmid for human transferrin receptor) have been previously described (gift of Dr. T. Mc Graw, Cornell University) [46] and were maintained in Ham's F-10 medium (Invitrogen) without or with 0.2 mg/ml of geneticin (Invitrogen) respectively. Each of the culture media containing L-glutamine described above was supplemented with 10% fetal bovine serum, and antibiotics penicillin (100 U/ml) and streptomycin (100 μg/ml).

### Confocal and electron microscopy

Approximately 10,000 cells/well of HeLa cells were plated in a 12-well tissue culture plate containing circular glass cover slips. After overnight incubation, the cells were exposed to either 10 μg/ml of Texas red-labeled transferrin (Invitrogen) or 20 μg/ml of CPV-VLPs or both (for co-localization studies) for 2 hrs at 37°C in media without serum. Following incubation the cells were washed 3 times with media and then fixed with ice-cold 4% paraformaldehyde in PBS (pH 7.4) for 10 mins. After fixing, the cells were washed 3 times with PBS and then treated for 10 mins in PBS containing 0.1% Triton X-100 and 1% bovine serum albumin (permeabilization buffer). The cells were exposed to rabbit anti-CPV antibodies (1:500) diluted in permeabilization buffer for 1 hr at room temperature. The cells were washed three times in PBS and exposed to Alexa-488 labeled goat anti-rabbit antibodies (Invitrogen) at a dilution of 1:2000 in permeabilization buffer for 30 mins at room temperature. The cover slips were washed three times with PBS then quickly with water prior to mounting with Vectashield hard set medium (Vector Laboratories, Burlingame, CA) on glass slides. The cells were examined with a Zeiss Axiovert Confocal microscope. For experiments with TRVb, TRVb1 and various tumor cells, each of the cell line was exposed to OG488-CPV-VLP under similar conditions as described above. Additionally, TRVb cells were treated with TOTO-3 (Invitrogen) for nuclear staining. Following fixation the cells were washed with PBS and directly visualized by confocal microscopy.

Transmission electron microscopic analyses of CPV-VLPs were performed by depositing 10 μl aliquots of sample onto 100-mesh carbon-coated copper grids for 2 minutes. The grids were then stained with 10 μl of 2% uranyl acetate and visualized under a Philips CM100 electron microscope.

## Authors' contributions

PS conceived the study and performed experiments. GD assisted with dye labeling, virus structural modeling and column chromatography analyses. AS assisted with the baculovirus expression system and virus purification. MM provided guidance with the experimental design and manuscript preparation. All authors read and approved the final manuscript.
